# STRUCTURAL BARRIERS TO UTILIZATION OF SUPPORTIVE SERVICES AMONG LATINO CAREGIVERS OF PEOPLE LIVING WITH DEMENTIA

**DOI:** 10.1093/geroni/igae098.0697

**Published:** 2024-12-31

**Authors:** Rafael Cardoso-Arias, Maggie Ramirez

**Affiliations:** University of Washington, Seattle, Washington, United States; University of Washington, Seattle, Washington, United States

## Abstract

Underutilization of supportive healthcare and social services may contribute to Latino caregivers’ higher risk of experiencing adverse health outcomes. The study objective was to understand structural barriers to utilization of supportive services among Latino caregivers of people living with dementia (PLWD). In Washington State, we interviewed 30 Latino caregivers of PLWD and 14 healthcare and social service providers. We applied deductive coding to the interview transcripts—relying on predefined codes representing the Cultural Treatment Adaptation Framework. We then examined excerpts coded under “Treatment Access/Entry.” Lastly, we inductively derived themes representing structural barriers to Latino caregivers’ utilization of supportive services. We identified structural barriers within the following three themes: 1) ethnic and language discordance between providers and Latino caregivers, 2) limited coordination between health systems and the broader network of providers in the community, and 3) digital health inequities.These interconnected themes underscore the importance of culturally competent, linguistically accessible care and technologically innovative approaches for Latino families. Healthcare systems often adhere to standards of care primarily designed for a majority population, which may fall short in meeting the unique needs and preferences of Latino communities and lead to disparities in access to and utilization of supportive services. Overall, the study findings can inform decision makers to adapt and adopt targeted interventions, policies, and practices, to increase and improve utilization of supportive services for Latino PLWD and their family caregivers.

